# Benchmarking microbial DNA enrichment protocols from human intestinal biopsies

**DOI:** 10.3389/fgene.2023.1184473

**Published:** 2023-04-26

**Authors:** Dmitrij Marchukov, Jiaqi Li, Pascal Juillerat, Benjamin Misselwitz, Bahtiyar Yilmaz

**Affiliations:** ^1^ University Hospital Zürich, University of Zürich, Zürich, Switzerland; ^2^Department of Visceral Surgery and Medicine, Bern University Hospital, University of Bern, Bern, Switzerland; ^3^ Maurice Müller Laboratories, Department for Biomedical Research, University of Bern, Bern, Switzerland; ^4^ Crohn’s and Colitis Center, Gastroenterologie Beaulieu, Lausanne, Switzerland

**Keywords:** gut micobiome, host DNA depletion, metagemonic, phyloseq, human small intestine, microbial enrichment

## Abstract

Shotgun metagenomic sequencing is a powerful tool for studying bacterial communities in their natural habitats or sites of infection, without the need for cultivation. However, low microbial signals in metagenomic sequencing can be overwhelmed by host DNA contamination, resulting in decreased sensitivity for microbial read detection. Several commercial kits and other methods have been developed to enrich bacterial sequences; however, these assays have not been tested extensively for human intestinal tissues yet. Therefore, the objective of this study was to assess the effectiveness of various wet-lab and software-based approaches for depleting host DNA from microbiome samples. Four different microbiome DNA enrichment methods, namely the NEBNext Microbiome DNA Enrichment kit, Molzym Ultra-Deep Microbiome Prep, QIAamp DNA Microbiome kit, and Zymo HostZERO microbial DNA kit, were evaluated, along with a software-controlled adaptive sampling (AS) approach by Oxford Nanopore Technologies (ONT) providing microbial signal enrichment by aborting unwanted host DNA sequencing. The NEBNext and QIAamp kits proved to be effective in shotgun metagenomic sequencing studies, as they efficiently reduced host DNA contamination, resulting in 24% and 28% bacterial DNA sequences, respectively, compared to <1% in the AllPrep controls. Additional optimization steps using further detergents and bead-beating steps improved the efficacy of less efficient protocols but not of the QIAamp kit. In contrast, ONT AS increased the overall number of bacterial reads resulting in a better bacterial metagenomic assembly with more bacterial contigs with greater completeness compared to non-AS approaches. Additionally, AS also allowed for the recovery of antimicrobial resistance markers and the identification of plasmids, demonstrating the potential utility of AS for targeted sequencing of microbial signals in complex samples with high amounts of host DNA. However, ONT AS resulted in relevant shifts in the observed bacterial abundance, including 2 to 5 times more *Escherichia coli* reads. Furthermore, a modest enrichment of *Bacteroides fragilis* and *Bacteroides thetaiotaomicron* was also observed with AS. Overall, this study provides insight into the efficacy and limitations of various methods for reducing host DNA contamination in human intestinal samples to improve the utility of metagenomic sequencing.

## 1 Introduction

The gut microbiota is a complex community of microorganisms living in the mammalian digestive tract (Martin et al., 2007; Hilt et al., 2014; Sender et al., 2016). The highly co-evolved mutualism between inhabitants on our body surfaces and the host immune system has promoted beneficial co-existence and interdependency over millions of years (Young, 2017). The role of the bacterial microbiota in maintaining homeostasis starts at birth and continues throughout life (Dominguez-Bello et al., 2010; Mueller et al., 2015). It is notably evident that viable gut microbiota is crucial for maintaining the host health status (Lloyd-Price et al., 2019), and this is in nearly everyone’s interests to keep the habitat and its distinctive niches healthy (Stecher et al., 2005; Smith et al., 2007; Hooper and Macpherson, 2010; Yilmaz et al., 2014; Macpherson et al., 2018; Uchimura et al., 2018; Lynch and Hsiao, 2019). The composition of the gut microbiota remains relatively stable over the years within individuals in the absence of major events such as medications or surgery. Over time, gut microbial strains undergo genetic changes *via* various mechanisms (e.g., mutations, horizontal and vertical gene transfer), and selection resulting in rapid adaption and/or long-term evolution of sub-strains. These processes can lead to positive and negative dynamic structural and functional changes in the gut, which in turn might also impact human health (Yilmaz et al., 2021).

Changes in the gut microbiota have been associated with a wide range of diseases, including inflammatory bowel diseases (IBD) (Lloyd-Price et al., 2019; Yilmaz et al., 2019), celiac diseases (Olivares et al., 2018), colorectal cancer (CRC) (Feng et al., 2015; Liang et al., 2017; Yu et al., 2017) (Cheng et al., 2020), chronic inflammation and metabolic diseases (Cox et al., 2014) such as obesity (Ley et al., 2005) (Backhed et al., 2004; Turnbaugh et al., 2006) and diabetes (Stewart et al., 2018; Zhou et al., 2022). However, studying the intestinal microbiome in the context of these diseases poses unique technical challenges. Identifying the diversity of gut microbiota using culture-based methods can be a laborious and time-consuming process that is often unable to capture the full range of microbial species present. However, some studies have attempted to address this limitation by using over 60 different culture conditions to isolate the most abundant taxa. In these studies, the researchers were able to successfully culture an average of 95% of the operational taxonomic units (OTUs) present at greater than 0.1% abundance in fecal samples. (Browne et al., 2016; Lau et al., 2016). In recent years, molecular-based approaches that do not rely on cultivation, such as 16 S rRNA gene sequencing and metagenomics, have brought a paradigm shift to our comprehension of the human microbiome’s involvement in health and disease. These methods enable a thorough examination of the microbial community, including the detection of previously un-cultivable bacteria and the evaluation of their functional potential (Loman et al., 2012). Although 16 S rRNA amplicon sequencing is an expeditious and cost-effective approach for identifying the taxonomic composition of a sample (Schriefer et al., 2018), it is insufficient for characterizing the functional landscape of the gut microbiome to answer inquiries regarding microbial activities (Franzosa et al., 2018). Therefore, alternative strategies, such as shotgun metagenomic approaches, are needed to investigate the functional potential of the microbiome (Qin et al., 2010).

Shotgun metagenomic sequencing allows the simultaneous analysis of all genetic material present in a sample, regardless of the organisms. This approach enables the identification of numerous genes and their variants, along with the reconstruction of enzymatic pathways, thereby providing valuable insights into the functional capabilities of the microbial community (Ranjan et al., 2016; Robinson et al., 2021). In addition, recent advancements in microbiome research have expanded our ability to investigate the microbiota’s functional and genetic profile in specific regions of the intestine, due to the development of bacterial profiling techniques that can be applied to biopsies rather than stool samples possible (Korem et al., 2015; Suez et al., 2018; Saffarian et al., 2019; Yilmaz et al., 2019; Zeevi et al., 2019; Yilmaz et al., 2022). This approach has been successfully utilized to unravel the molecular and cellular mechanisms underlying gut-associated diseases. For instance, a study conducted by Franzosa et al. utilized shotgun metagenomic sequencing of colonic biopsies to identify gene-level differences in the microbial community between patients with Crohn’s disease and healthy individuals (Franzosa et al., 2019). This approach revealed that Crohn’s disease was associated with significant alterations in bacterial metabolic pathways, including amino acid metabolism, energy production, and xenobiotic biodegradation. Furthermore, the use of biopsy-based bacterial profiling has allowed for a better understanding of the microbial communities’ spatial organization in the intestine, with studies showing differences in microbial composition and diversity across various intestinal regions, including the duodenum, jejunum, ileum, and colon. For instance, a study by Leite et al. (2020) using 16 S rRNA gene sequencing of duodenal biopsies from healthy individuals revealed a distinct microbial community structure compared to that observed in fecal samples, highlighting the importance of analyzing specific regions of the intestine to gain a more comprehensive understanding of the microbiota’s functional and genetic profile. Therefore, the use of biopsy-based bacterial profiling has provided a promising avenue for investigating the molecular and cellular mechanisms underlying gut-associated diseases and holds great potential for future microbiome research.

Bacterial metagenomic sequencing requires a sufficient abundance of microbial DNA without large-scale host DNA contaminations. However, biopsies or whole-tissue isolates contain large bulks of host DNA, vastly outnumbering microbial DNA (de Albuquerque et al., 2022). This phenomenon is not limited to intestinal biopsies. We have recently conducted a study with ileostomy patients, where we identified highly dynamic components of the microbiota present in the small intestine. These components were found to be highly responsive to dietary changes introduced after an overnight fast. (Yilmaz et al., 2022). The ratio of microbial/host DNA oscillates in accordance with fasting and feeding, and the appearance and disappearance of microbial sub-strains were also strongly associated with the provision of nutrition. This results in a higher ratio of host/microbe DNA in the fasting state, while the introduction of food leads to blooming in bacterial populations and a lower ratio of host/microbe DNA (Yilmaz et al., 2022). In this type of situation, characterizing the functional and genetic profile of low-abundance bacteria in microbiome samples can be a difficult task, particularly if the sample is contaminated with host DNA. To overcome this challenge, it is essential to perform host DNA depletion as the first step before conducting deep shotgun metagenomic sequencing. By removing host DNA upstream of sequencing, it is possible to increase the detection of low-abundance bacteria, which would otherwise remain undetected. Nevertheless, the optimal approach to achieve host DNA depletion remains undetermined.

To reduce host DNA content and increase the yield of microbiota DNA prior to sequencing (Heravi et al., 2020), several commercial kits and general laboratory methods have been developed over the past few years. Some of them have already proven useful in enriching microbial DNA from liquid samples such as saliva (Marotz et al., 2018), blood (Feehery et al., 2013), sonicated fluid from prosthetic joint components (Thoendel et al., 2016), human milk (Yap et al., 2020), and cerebrospinal fluid (Hasan et al., 2016; Ji et al., 2020), but also from solid materials such as human breast tissue (Costantini et al., 2018), and tissue from an infected diabetic foot (Heravi et al., 2020). Surprisingly, to the best of our knowledge, commercially available kits for the depletion of host DNA from intestinal tissues have not been systematically compared or tested, except for a single study that developed a new technique to address this challenge. In this study, researchers optimized the sample lysis step by incorporating additional detergents and bead-beating protocols to achieve an efficient host DNA depletion (Bruggeling et al., 2021). The method was demonstrated to be relatively effective in reducing host DNA contamination in human fecal and mucosal samples. Notably, the approach resulted in higher bacterial read yield and a more accurate representation of the microbial community compared to the commercially available kits.

The present study aimed to evaluate the effectiveness of host DNA depletion methods, including commercially available kits, a laboratory-optimized protocol, and the software-controlled enrichment approach of Oxford Nanopore, in enhancing bacterial DNA yields from human intestinal biopsy samples. Our main objective was to enhance bacterial DNA yields from human intestinal biopsy samples, and the findings highlight the limitations of existing microbial DNA enrichment tools and the potential impact of different enrichment methods on the identification of bacterial groups. Despite the challenges of host DNA depletion from intestinal biopsies, we were able to increase the proportion of bacterial DNA to 30%–45% of total DNA in some cases. We also observed that different microbial enrichment methods could lead to shifts in the proportion of identified bacteria groups in each sample. Interestingly, we observed that the software-controlled enrichment approach of Oxford Nanopore increased the base pair numbers associated with bacterial DNA allowing to assemble the genome metagenomically but did not significantly increase the percentage of bacterial reads compared to commercially available kits. Our study provides valuable insights into the effectiveness of different microbial DNA enrichment methods and highlights the potential for the software-controlled enrichment approach of Oxford Nanopore to improve bacterial DNA yield and enable the identification of rare intestinal bacteria.

## 2 Materials and methods

### 2.1 Sample collection and ethics statement

To evaluate the performance of four host DNA depletion kits and one total DNA extraction control kit, we collected endoscopic biopsies (∼2 mm) from 10 subjects, with five biopsies per subject. Additionally, we included a second test group comprising 10 subjects to assess the effectiveness of a non-commercial method (Bruggeling et al., 2021) in combination with two commercial kits. In this group, three biopsies per subject were collected. The procedures used in this study included total DNA extraction, microbial DNA enrichment, and two variations of the published laboratory-optimized depletion method (Bruggeling et al., 2021).

The Bern Human Intestinal Community project was approved by the Bern Cantonal Ethics Commission (Ref: KEK-BE: 251/14) with signed informed consent obtained from all participants. Additionally, the bowel cleansing study was approved by ethical approval number 336/2014. Biopsy samples were collected from subjects registered for a screening ileo-colonoscopy without any gastrointestinal symptoms and without functional intestinal symptoms and negative results in all additional workups. The cohort comprised of 15 males and 5 females within the age range of 40–60 years old. It is noteworthy that none of the participants had taken antibiotics or any regular medications for the 6 months preceding the sampling. The licensed gastroenterologists collected biopsy samples and clinical data of all healthy subjects. Colonic biopsies were initially collected into 2 mL microfuge tubes containing 500 µL RNAlater (Sigma-Aldrich) and stored at −20°C until DNA extraction.

Structured clinical metadata were prospectively gathered based on pre-determined standards, documented electronically using Research Electronic Data Capture (REDCap) (Harris et al., 2009) and handled in R (http://www.r-project.org) using the *xlsx* and *data. frame* packages. Assessment of the microbiota composition from intestinal biopsies was then analyzed according to numerous parameters such age, sex, and sampling location. Statistical analyses were performed using Student’s t-test, Wilcoxon’s rank sum test, and Pearson’s chi-squared test to assess differences between groups.

### 2.2 Microbial DNA extraction and host DNA depletion

**FIGURE 1:**
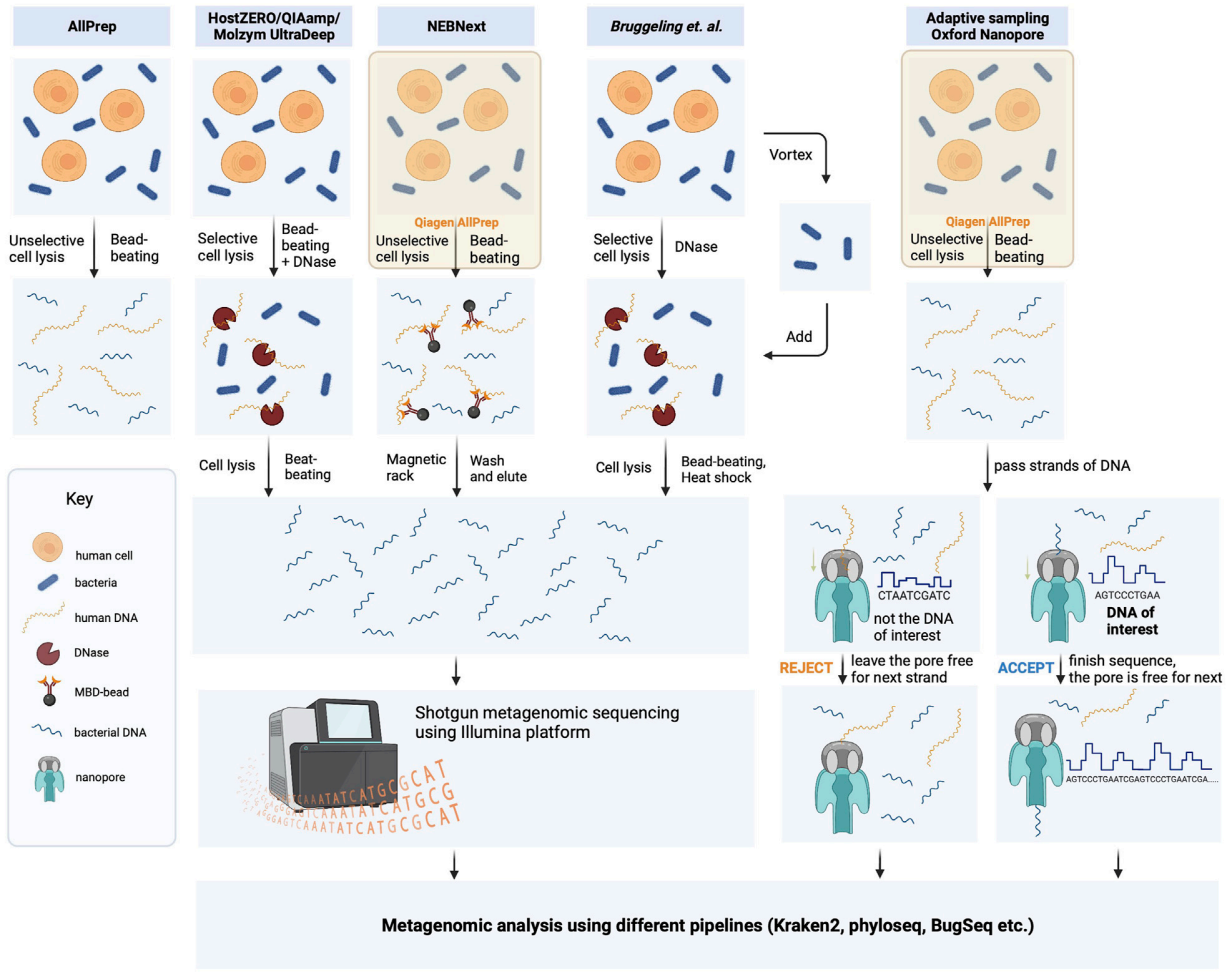
Schematic representation of DNA isolation protocol strategies used in this study. The bacterial DNA was extracted from biopsies using five different methods, which included protocols provided by the manufacturers and the laboratory-optimized protocol developed by Bruggeling and colleagues. (Bruggeling et al., 2021). These methods comprised unselective cell lysis kits (Qiagen AllPrep and NEBNext) and selective cell lysis kits (HostZero/QIAamp Microbiome and Molyzm UltraDeep), with or without microbiome enrichment. The resulting DNA samples were then sequenced using Illumina NovaSeq 6,000 in 150 bp paired-end mode. Additionally, DNA samples obtained through the Qiagen AllPrep DNA/RNA extraction kit were also analyzed using adaptive sequencing technologies from Oxford Nanopore.

Microbial DNA extraction is a critical step in metagenomic studies that can significantly impact downstream analyses. To ensure reproducibility and minimize bias, we followed the manufacturer’s instructions for DNA extraction using commercially available kits. Specifically, we employed the kits shown in Figure 1, which have been extensively validated and optimized for high yield and purity of DNA from a diverse range of microbial samples. However, these kits and computer-based approaches have not been extensively tested for human intestinal biopsies.

#### 2.2.1 Total DNA/RNA extraction

Total DNA was isolated using AllPrep DNA/RNA Mini Kit (Qiagen) as described before (Yilmaz et al., 2019). 600 µL of RLT Plus Buffer containing 6 µL beta-mercaptoethanol and a 3 mm bead were added into the tube. Biopsies were homogenized by the Retsch Tissue Lyser (Qiagen) at 30/frequency for 5 min. Supernatants were transferred into AllPrep DNA mini spin column and centrifuged at 9000 g for 30 s. DNA attached to spin columns was subjected to clean-up using 500 μL of Buffer AW1 and AW2 afterwards. As a last step, DNA samples were eluted with 30 μL nuclease-free water into 1.5 mL microfuge tubes and stored (−20°C) until proceeding with downstream steps. The concentration and purity of the isolated DNA samples were evaluated by NanoDrop^®^ (Thermo Scientific). Of note, RNA was extracted following the protocol instructions even though not used in our study.

##### 2.2.1.1 HostZERO microbial DNA kit

This kit initially applies the physical homogenization of tissue samples with bead-beating, followed by selective chemical lysis of eukaryotic host cells using the Host Depletion Solution, with the intention to keep microbial cells intact. In the host depletion part of the protocol, 200 μL Host Depletion was added to each sample and incubated at room temperature for 15min. Following centrifugation (10000*g*, 5min), the supernatant was discarded, and 100 μL of Microbial Selection Buffer and 1 μL of Microbial Selection Enzyme were added to each tube for incubation at 37°C for 30 min. To enhance the depletion of host DNA, 20 μL of Proteinase K were added to the sample and incubated at 55°C for 30 min 100 μL of DNA/RNA Shield™ (2X Concentrate) was added. For microbial DNA isolation, each sample was treated with ZR BashingBead™ Lysis Tube and 750 μL of ZymoBIOMICS^®^ Lysis Solution. The Retsch Tissue Lyser (Qiagen) was used for 5 min at a frequency of 30/min. Next, 400 μL of supernatant was transferred to another collection tube. After adding 1200 μL of ZymoBIOMICS^®^ DNA Binding Buffer to the tubes, mixing was done by thoroughly pipetting the entire volume up and down five times. Each sample was then transferred to the Zymo-SpinTM IC-Z Column, and ZymoBIOMICS^®^ DNA Wash Buffer 1 and Wash Buffer 2 were applied in the washing step. After the final centrifugation (10000g, 2 min) of the washing steps, 30 μL DNase-free water was applied to the center of the Zymo-SpinTM IC-Z Column. DNA was eluted by centrifugation at 10000g for 1 min and stored at −20°C.

#### 2.2.2 QIAamp DNA microbiome kit

This kit also works based on the principle of lysing host cells first and depleting host DNA enzymatically while keeping microbial cells intact for the downstream microbial DNA extraction process. Briefly, 500 μL of Buffer AHL was added to each tube, followed by incubation at room temperature for 30 min. After a centrifugation step at 10000 g for 10 min, the supernatant was removed. 190μL of Buffer RDD and 2.5 μL of Benzonase were added into each tube and incubated at 37°C for 30 min on a thermomixer with shaking at 600rpm) After the addition of 20 μL Proteinase K, samples were again incubated at 56°C for 30 min on a thermomixer (600rpm). Then, 200 μL Buffer ATL was added to each sample and transferred into Pathogen Lysis Tube L. After lysis, samples were heated to 95°C for 5min. Following centrifugation, 40 μL Proteinase K was added to each sample, vortexed, and incubated at 56°C for 30 min. Next, 200 μL Buffer APL was added to each tube followed by incubation at 70°C for 10 min. Afterwards, 200 μL ethanol was added to the lysate and mixed by pulse-vortexing for 15–30 s. 700 μL of the mixture was then transferred into the QIAamp UCP Mini spin column. Washing steps using 500 μL of Buffer AW1 and AW2 were done following the instructions of the protocol. After the final centrifugation of the washing steps, 30 μL DNase-free water was applied to the center of the membrane. DNA was eluted by centrifuging at 10000 g for 1 min and stored at −20°C.

#### 2.2.3 Molzym ultra-deep microbiome prep kit

This kit utilizes a combination of mechanical and enzymatic lysis to effectively release DNA from cells and includes a bead-beating step for efficient cell lysis. This allows the degradation of free-floating and human DNA and isolates the genomic DNA of microbes. The DNA was then purified using silica-based spin column technology. To reduce the interference of host DNA in microbial DNA sequencing, this method selectively lyzed human cells using CM buffer, followed by degradation of host-released DNA using human DNase (MolDNase B), leaving bacterial cells intact. Bacterial cells were then concentrated by centrifugation, and DNA was extracted using enzymes that specifically target bacterial cell walls. To ensure consistency and reproducibility, all steps of the protocol were conducted precisely following the manufacturer’s instructions.

#### 2.2.4 NEBNext microbiome DNA enrichment kit

In contrast to the three kits described above, the starting material of this kit is total DNA. The NEBNext Microbiome DNA enrichment kit (New England Biolabs) contains magnetic beads that selectively bind to the CpG-methylated host DNA (Feehery et al., 2013). This step facilitates the enrichment of bacterial DNA and the depletion of host DNA. Briefly, total DNA was extracted using the AllPrep DNA/RNA Mini Kit (Qiagen) using a slow bead beating step at a frequency of 10/min for 5 min. Host methylated DNA was captured using 160 μL of MBD2-Fc-bound magnetic beads and prepared according to the manufacturer’s instructions. The undiluted bind/wash Buffer (5X) was added to make the final concentration 1X and tubes were agitated by rotating at room temperature for 15 min. Each tube was then placed on the magnetic rack for 5 min until the beads were collected on the wall of the tube and the solution was clear. The supernatant was removed without disturbing the beads and transferred to a clean microcentrifuge tube. This supernatant contains the target microbial DNA. Afterwards, the ethanol precipitation protocol was followed to elute captured host DNA. 2.5X pure ethanol was added to each sample and then incubated on ice for 10 min. Afterwards, ethanol was removed by a centrifugation step at 16000 g for 30 min and pellets were air-dried.

#### 2.2.5 Host DNA depletion by the method proposed by *Bruggeling et al.*

Bruggeling et al. (2021) proposed a bacterial DNA isolation method optimized for the human gut biopsy tissue. We followed the protocol described in this manuscript. Briefly, bacteria loosely bound to the surface of the biopsy were separated by vortexing and transferred to another microfuge tube. 20 μL proteinase K and 0.0125% saponin were then used for digestion and lysis of human cells while keeping bacterial cells intact. The resulting cell suspension thus contained lyzed human cells and intact bacterial cells. Next, DNase treatment was applied for human DNA depletion. In the end, each sample had reduced human DNA content and intact bacteria. Bacterial cells were then lyzed by utilizing a specialized bead-beating protocol, using 0.5 KU/mL mutanolysin (Sigma) and a brief heat shock to ensure susceptibility for mechanical lysis.

We altered the protocol slightly to increase the yield of microbial DNA: In step 3 of the original protocol, instead of vortexing the tubes for 5 min samples were put in a bead-beater at 10 Hz for 5 min without beads. Further, instead of adding 2 μL TurboDNAse in 10x Turbo DNAse buffer, we used 2.5 μL DNase I in Buffer RDD of the RNase Free DNase Set by Qiagen. Moreover, in the original protocol, 20 μL mutanolysin per sample was used, but we reduced it to half. HostZERO Microbial DNA Kit and the QIAamp DNA Microbiome Kit were tested separately for bead-beating and microbial DNA extraction.

#### 2.2.6 Library preparation and shotgun metagenomic sequencing

Due to a low DNA concentration in some groups of samples, we prepared the libraries using the Nextera XT kit, which requires a minimum of 2 ng DNA as the starting material. DNA libraries were prepared according to the Nextera DNA Library Prep (Illumina) as instructed in the manufacturer’s protocol and sequenced on NovaSeq 6,000 (Illumina, 150bp, PE mode). The metaWRAP-Read_qc module was applied to filter out the human genome‒contaminated reads, remove adaptor sequences, and low-quality reads, and produce quality reports for each of the sequenced samples prior to the microbial abundance estimation (Uritskiy et al., 2018). This pipeline contains the FASTQC (Andrews, 2015) and the BMTagger modules (Rotmistrovsky, 2011).

Every sample was subjected to Illumina sequencing, while an additional 5 samples from the initial group that were extracted using AllPrep later underwent ONT sequencing as well.

Before conducting any subsequent diversity and taxonomy analysis, the read counts of each sample were divided by the total number of reads in that sample, using the library size normalization method. The taxonomy profile was assessed using the Kraken2 pipeline with a custom RefSeq database following the developer’s guideline (Wood et al., 2019) and the Kraken report was generated in https://github.com/DerrickWood/kraken2/blob/master/docs/MANUAL.markdown#custom-databases. To generate the CustomDB taxonomy directory with the necessary information, we executed the kraken2-build command with the "--download-taxonomy” option. This allowed us to obtain the accession number to taxon maps, taxonomic names, and tree information from NCBI. However, the information was limited to complete genomes of archaea, bacteria, fungi, plants, protozoa, and virus. For taxonomy classifications, we utilized Kraken2, with the following command line serving as a representative example: *kraken2 --use-names--db/home/ubelix/dbmr/terziev/CustomDB--fastq-input--report-zero-counts--confidence 0.1 --threads 12 --minimum-base-quality 0 --paired--gzip-compressed {input_1. fastq.gz} {input_2. fastq.gz} -- output {output.reads} --report {output.report} > input. kraken.*

#### 2.2.7 Library preparation and sequencing with Oxford Nanopore Technologies (ONT)

1 µg of high molecular weight DNA samples obtained using AllPrep DNA/RNA kit were used for Oxford Nanopore adaptive sequencing. Library preparation was performed using the SQK-LSK110 kit (Oxford Nanopore Technologies, Oxford) following the genomic DNA ligation protocol (https://community.nanoporetech.com/protocols/genomic-dna-by-ligation-sqk-lsk110/). Finally, the libraries were loaded separately onto different Nanopore R9.4.1 flow cells (FLO-MIN106), one for sequencing with Adaptive sampling (AS) and one for control sequencing. Both flow cells were run simultaneously on a GridION X5 device (MinKNOW version 21.11.7; Guppy 5.1.13; Oxford Nanopore Technologies) [ONT]) for up to 72 h.

#### 2.2.8 Bioinformatic analysis of Oxford Nanopore Technologies (ONT) adaptive sampling (AS) sequencing data

The output of AS sequencing runs consists of nanopore reads in a FASTQ format, accompanied by a csv file that lists the classification of each read made by the ReadUntil API (https://github.com/nanoporetech/read_until_api) available in the MinKNOW interface on the GridION machine. This classification is based on read matching to the user-provided reference sequence(s), as follows: Under a “depletion” AS runs as follows: the initial 400–600 bases of a strand that translocate through a given pore are used to classify the reads by the ONT’s ReadUntil API. Each read that passed through the nanopore was aligned to the human reference genome while it was being sequenced. The alignment occurred at intervals of several bases, and three types of decisions were made: 1) “no_decision”—the read was continued and realigned to the reference(s) after several bases (“no decision”), 2) “stop_receiving”—the read was fully sequenced and accepted (“accepted”), and 3) “unblock”—the sequencing was immediately terminated, and the read was rejected. In the “rejected” case, the voltage is reversed at the pore level and the DNA will be expelled from the pore, preventing further sequencing Payne et al. (2021).

Taxonomy classification and quality control analysis of long-read sequences were performed using the BugSeq workflow which uses a combination of tools and databases to classify reads into taxonomic groups and also identify potential contaminant sequences (Fan et al., 2021; Chandrakumar et al., 2022). Briefly, the reads were quality-controlled using *fastp* with a minimum read length of 100bp and minimum average read quality of Phred 7 (Chen et al., 2018). Then, the reads were mapped to BugSeq’s curated database containing microbial sequences, the human genome, and contaminants using minimap2 in “map-ont” mode with the “-a” flag (Li, 2018). The alignments were then reassigned using *Pathoscope* based on a Bayesian statistical framework and the lowest common ancestor of alignments was taken (Francis et al., 2013). Finally, the lowest common ancestor of the reassigned reads was calculated using *Recentrifuge* (Marti, 2019). The output obtained with this pipeline was saved in csv files which were then used to prepare the corresponding tables and figures in this study using GraphPad Prism Version 9.5.1.

#### 2.2.9 Statistical analysis

All statistical analyses were performed using R version 3.6.1 or Prism 9 (GraphPad Software, San Diego, CA). Differences between the groups after library size normalization were evaluated using one-way ANOVA (parametric), followed by Tukey’s honest significant difference test or the two-stage step-up method of Benjamini, Krieger, and Yekutieli, as a *post hoc* test. The effect size was calculated using Cohen’s d in Excel using the following formula = ABS (AVERAGE (group1) - AVERAGE (group2))/(SQRT (((COUNT (group1)—1) * STDEV. S (group1, group2)^2 + (COUNT (group2) - 1) * STDEV. S (group2,group1)^2)/(COUNT (group1) + COUNT (group2)—2))).

The computation of alpha and beta diversity was carried out using different metrics, such as the Shannon index for alpha diversity and Aitchison distance for beta diversity. These computations were performed using the *phyloseq* package in R. (McMurdie and Holmes, 2013; Callahan et al., 2016). Statistical analyses were performed using Mann-Whitney U tests for alpha diversity and Adonis (PERMANOVA) for beta diversity with pairwise comparison (Benjamini-Hochberg false discovery rate correction) using *pairwiseAdonis R* package to confirm the strength (McMurdie and Holmes, 2013; Callahan et al., 2016). Microbial changes were tested using multivariate analysis by linear models (MaAsLin2) R package (Morgan et al., 2012; Mallick et al., 2021). Differences of *p* < 0.05 or adj-p < 0.05 were considered significant in all statistical analyses.

## 3 Results

### 3.1 Commercial kits can enrich the microbial DNA but cannot entirely deplete host DNA from intestinal biopsy samples

Depletion of host DNA from biopsies is crucial for identifying the bacteria present in a specific region of the human intestine, as well as for characterizing the genetic features of these bacteria *via* metagenomic analysis. We evaluated several commercially available DNA depletion kits, including the HostZERO Microbial DNA kit, Molzym Ultra-Deep Microbiome Prep Kit, NEBNext Microbiome DNA Enrichment Kit, and QIAamp DNA Microbiome Kit, to assess their suitability for extracting DNA from intestinal biopsy samples. These kits employ different strategies for lysing host cells and enzymatically degrading host DNA, except for the NEBNext kit, which utilizes a different approach by selectively removing CpG-methylated host DNA from total DNA extracted from AllPrep DNA/RNA Mini Kit (Figure 1).

The concentration and purity of the isolated DNA samples were analyzed by NanoDrop^®^ (Thermo Scientific). The purity with different extraction kits was in an acceptable range with a 260 nm/280 nm absorption ratio varying between 1.75 and 2.10 (Table 1). In addition, the DNA was not fragmented with any of the extraction kits, as indicated by a Bioanalyzer 2,100 measured DNA integrity number (DIN) between 7.25 and 8.55. Additional tests indicated some impurities with Molzym, QIAamp Microbiome, and HostZERO tests revealing relatively low 260/230 ratios. This low ratio is likely due to traces of residual guanidine from the lysis buffer used in column-based kits. However, such impurities typically do not affect downstream sequencing analysis, as stated by the manufacturers’ application note. Notably, seven samples were excluded from the analysis due to low DNA yield, leaving 43 samples for further analysis.

**TABLE 1:** Quality control of DNA extracted by the multiple kits from endoscopic biopsy samples. DNA concentration, purity, and DNA integrity number (DIN) are recorded for each sample. The average values with standard deviations (±) are shown for each category.

*Extraction Method*	*Sample Size*	DNA concentration (ng/μL)		260/280 ratio		*260/230 ratio*	*DN*
*AllPrep*	10	173.28 ± 204.3		1.90 ± 0.01		2.35 ± 0.05	8.25 ± 0.05
*NEBNext*	6	19.4 ± 6.8		2.01 ± 0.30		2.11 ± 0.30	8.35 ± 0.15
*HostZero*	8	1.3 ± 1.1		1.75 ± 0.21		0.39 ± 0.21	7.55 ± 0.13
*Moyzm*	10	53.23 ± 46.1		2.10 ± 0.25		0.85 ± 0.05	8.55 ± 0.35
*QIAamp*	9	2.92 ± 1.9		2.07 ± 0.22		0.47 ± 0.11	7.25 ± 0.42
*AllPrep2*	9	285.4 ± 185.1		1.85 ± 0.05		2.15 ± 0.10	8.15 ± 0.11
*QIAamp + Lab-optimized*	8	15.48 ± 35.6		2.25 ± 0.27		1.25 ± 0.55	8.25 ± 0.15
*HostZero + Lab-optimized*	6	1.8 ± 1.7		1.85 ± 0.40		0.49 ± 0.01	7.69 ± 0.13

Metagenomic sequencing using the 150 bp PE mode on an Illumina NovaSeq platform yielded a total of 1′002′860′693 reads from 43 extracted DNA samples (Supplementary Table S1). Of these reads, only 97′015′448 were assigned to the microbial portion using the Kraken2 pipeline with a custom dataset containing microbial and human databases (Supplementary Table S1). Notably, 66′469′799 reads failed to match any of the databases and hence they were not further analyzed. These reads are likely derived from plant DNA in line with our recent study, which showed that Kraken2 could effectively identify plant DNA even in samples with abundant host material (Yilmaz et al., 2022). Since plant DNA was not the focus of this study, these reads were not further analyzed.

**FIGURE 2:**
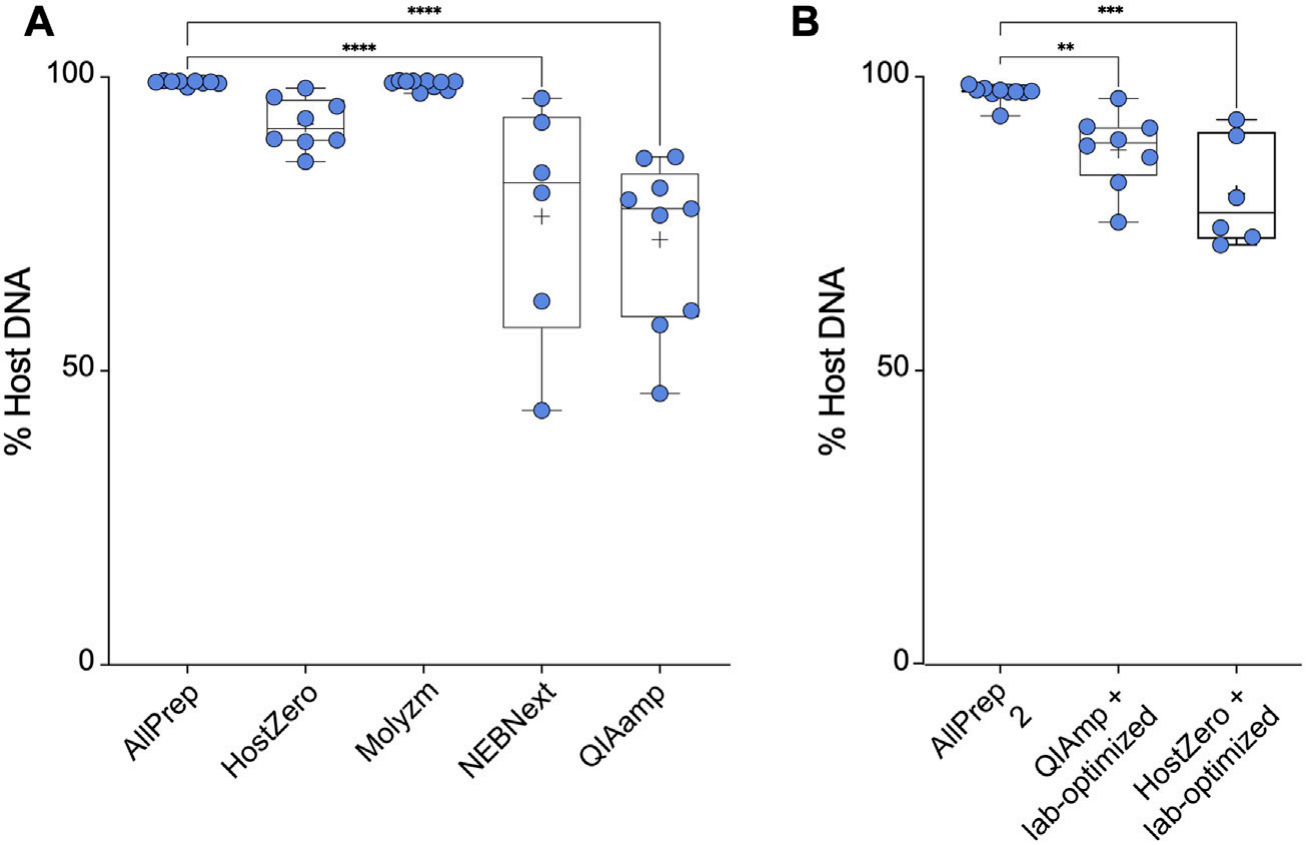
Microbial enrichment kits reduce host DNA in extracted DNA samples. **(A)** Percentage of host DNA in samples prepared using different microbiome DNA enrichment methods was compared to the percentage of host DNA extracted using a total DNA extraction kit (AllPrep). **(B)** As in **(A)**, only the methods of the indicated microbiome DNA enrichment kits were modified as described by *Bruggeling et al.* Asterisks for *p*-values: *p < 0.05, **p < 0.01, ***p < 0.001 and ****p < 0.001. The Cohen’s d value for each comparison is as follows: HostZero versus AllPrep (d = 1.22), Molysis versus AllPrep (d = 0.44), NEBNext versus AllPrep (d = 1.36), QIAamp versus AllPrep (d = 1.72), QIAamp + Lab-optimized versus AllPrep (d = 1.52) and HostZero + Lab-optimized versus AllPrep (d = 1.79).

The most effective kit for microbial DNA enrichment was the QIAamp DNA microbiome kit, which enriched the microbial DNA population to ∼28% on average. Similarly, the NEBNext kit enriched the microbial DNA population, yielding an average of ∼24% microbial DNA (Figure 2A). This demonstrates that some of the commercially available microbial DNA enrichment kits are effective in reducing host DNA from intestinal tissue samples, specifically the NEBNext and QIAamp methods. However, not all extraction kits were able to enrich microbial DNA. Based on the host DNA/microbial DNA ratios, we concluded that the HostZero and Molzym kits did not affect the host DNA content: Extraction with the control AllPrep kit yielded on average ∼1.0% microbial DNA, whereas the Molyzm and HostZero kits resulted in an average of only ∼0.2% and ∼7.0% bacterial DNA, respectively (Figure 2A).

We previously demonstrated that investigating SNPs and structural variants in the most abundant taxa requires over ∼90% of microbial DNA in a given sample with more than 50 million reads (Yilmaz et al., 2022). However, as shown above, none of the commercial kits was able to reduce the host DNA content but did not achieve the desired purity of ≥90% (Figure 2A). Therefore, we attempted to optimize the commercial kit protocols (laboratory-optimized protocol) by adding additional vortexing steps as well as saponin incubation steps as described by Bruggeling et al. (2021) before performing all extraction steps of the respective kit. We tested this modification for the QIAamp and HostZero kits and compared the results to those obtained using the AllPrep kit. Our results showed that the AllPrep kit with optimization yielded on average ∼3.0% microbial DNA, slightly higher than without the optimization steps (Figure 2). While for the QIAamp kit, the optimization did not significantly enrich microbial DNA (∼13%), the HostZero kit with laboratory-based optimization increased microbial DNA yield to up to 20% (Figure 2B). Overall, these results suggest that while commercial kits can reduce host DNA contents in samples, they may not be sufficient for advanced microbial genetic analysis. However, since simple additional steps such as extra vortexing and saponin supplementation improved the efficacy, suggesting that there may be room for further improvement in microbial DNA enrichment protocols.

### 3.2 Impact of host depletion with different extraction kits on microbial community abundance and composition

**FIGURE 3:**
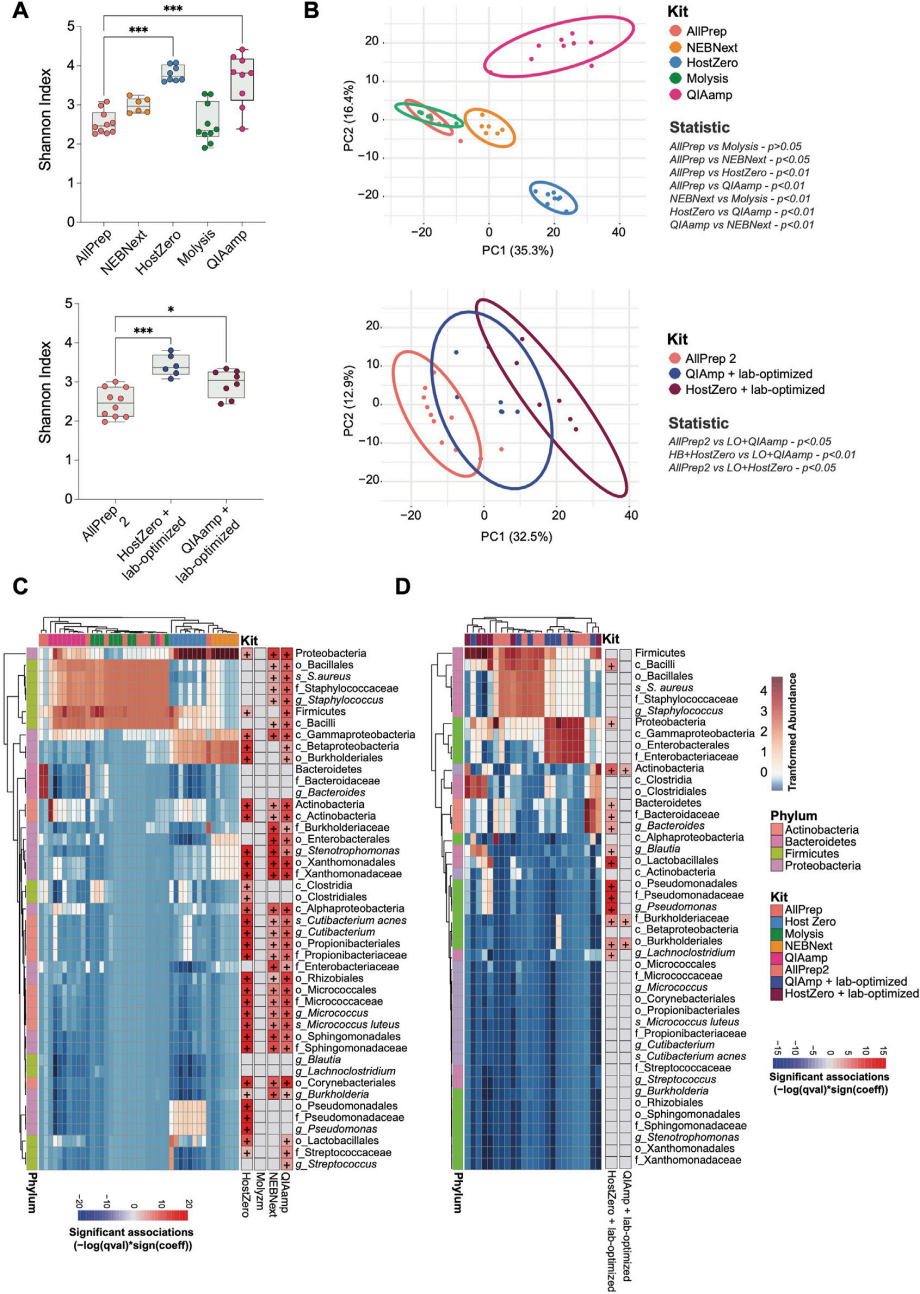
Shifts in the observed bacterial composition induced by various host depletion kits. The bacterial DNA of human intestinal biopsies was analyzed by shotgun metagenomic analysis. **(A)** Alpha diversity between different host depletion kits was measured using the Shannon index and presented on box-and-whisker plots displaying quartiles, range, and standard deviations. **(B)** Differences in microbial composition between the groups were analyzed with Aitchison distance. Ellipsoids represent the 95% confidence interval of the position of each group. The non-parametric Mann-Whitney U-test and the Adonis test were used to determine statistically significant differences between groups regarding alpha diversity **(A)** and beta diversity **(B)**, respectively. **(C)** A heatmap was generated to show the relative abundance of each taxon that differed between host depletion kits compared to the standard AllPrep kit. **(D)** A similar heatmap was generated for host depletion kits combined with the lab-optimized approach compared to the standard AllPrep kit. A *p*-value less than 0.05 was considered significant, and significant taxa are shown on the right panel of each heatmap with the color representing associations calculated using -log (q-value)*sign (coefficient), where "+" represents an adj-p-value <0.05. Asterisks for *p*-values: *p < 0.05, **p < 0.01, ***p < 0.001 and ****p < 0.001. The Cohen’s d value for each comparison is as follows for **(A)**: HostZero versus AllPrep (d = 1.33), Molysis versus AllPrep (d = 1.82), NEBNext versus AllPrep (d = 0.03), QIAamp versus AllPrep (d = 1.47), QIAamp + Lab-optimized versus AllPrep (d = 1.15) and HostZero + Lab-optimized versus AllPrep (d = 1.65).

The level of microbial DNA enrichment differs among these kits. Although none of these enrichments were adequate for conducting a comprehensive genetic profile of the most prevalent taxa in the gut, we next investigated the similarity of the microbial community composition in samples extracted with different kits (Figure 3). The microbiota of the HostZero and QIAamp kits show a greater diversity of species, even with the laboratory-optimized optimization protocol (Figure 3A). Further, relative composition differences of the intestinal microbiota were found with NEBNext, HostZero and QIAamp kits compared to standard AllPrep kit, assessed by PCA with Aitchison distance (*p* < 0.01) (Figure 3B). These findings remained robust, also when the laboratory-optimized protocol was utilized (*p* < 0.05). Furthermore, our analysis of all samples collectively revealed a positive correlation between microbial enrichment and alpha diversity, with statistical significance observed (*p* < 0.05) (Supplementary Figure S1A). Additionally, Supplementary Figure S1B reveals a clustering pattern among the samples based on their microbial DNA abundance.

Variations in host depletion methods affected certain phyla and families of bacteria much stronger than others. Specifically, we observed significant changes in the Actinobacteria, Bacteroidetes, Firmicutes, and Proteobacteria phyla (Figures 3C, D; Supplementary Table S2). Within the Proteobacteria phylum, the Pseudomonadales and Enterobacterales orders, as well as the Xanthomonadaceae (*Stenotrophomonas* genus), Burkholderiaceae, Enterobacteriaceae, and Sphingomonadaceae families, were more enriched after subjecting to extraction using HostZero, NEBNext, and QIAamp kits compared to the standard AllPrep kit. Moreover, HostZero and QIAamp kits yielded in an increase in the relative abundance of the Micrococcaceae family (*Micrococcus luteus* species) from the Actinobacteria phylum, as well as the Staphylococcaceae family (*Staphylococcus aureus* species) and Streptococcaceae family (*Streptococcus* genus) of the Firmicutes phylum, even though to a lesser extent (Figure 3C).

Taxa shifts could also be demonstrated when the lab-based optimization protocol was applied before the usage of the HostZero and QIAamp kits (Figure 3D). Changes in taxa observed with the HostZero lab-optimized approach were similar to those without the lab-optimized protocol shown in Figure 3C. Furthermore, under these conditions, the HostZero kit led to the enrichment of the Bacteroidaceae family (*Bacteroides* genus) of Bacteroidetes and the Lachnospiraceae family (*Lachnoclostridium* and *Blautia* genera) of Firmicutes, but the QIAamp kit did not show this effect (Figure 3D). These findings suggest that shifts in the observed bacterial compositional due to the usage of different host DNA depletion kits affect only a relatively small number of taxa, primarily from the Proteobacteria and Firmicutes phyla and less from Actinobacteria and Bacteroidetes.

### 3.3 Bacterial sequence enrichment in human intestinal samples using ONT adaptive sampling

Our findings showed that the ability to detect bacterial strains in intestinal biopsies with high levels of host DNA (>98%) is rather insufficient or ineffective with the existing wet-lab procedures of selective lysis of host and microbial cells or selective removal of CpG-methylated host DNA. Therefore, we carried out an alternative to lab-based depletion or enrichment approaches which is based on a software-controlled enrichment method by depletion of unwanted DNA during sequencing with providing a target DNA sequence (Martin et al., 2022) and it is called adaptive sampling (AS). ONT AS method allows the currently being sequenced DNA fragment in a given pore to be compared instantly with provided references to determine whether to sequence the DNA further (accepted or undecided) or reject it from the pore (rejected), which increases the sequencing capacity for molecules of interest (Loose et al., 2016).

**FIGURE 4:**
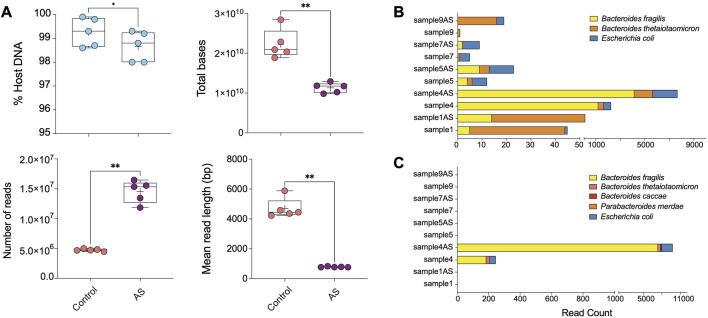
ONT AS enriches sequencing yield and the number of sequenced bacterial reads from biopsies. **(A)** The comparison between the percentage of host DNA in samples sequenced with (AS, *n* = 5) and without (Control, *n* = 5) the adaptive sampling approach was based on multiple parameters, including total bases, the total number of reads, and the mean read length (base pair). The bar plots show the most abundant enriched taxa classified from **(B)** reads and **(C)** metagenomic assemblies for samples sequenced on ONT with and without adaptive sampling. Samples ending with “AS” annotation are sequenced with the ONT AS approach, and the rest are without this approach. Asterisks for *p*-values: *p < 0.05, **p < 0.01, ***p < 0.001, and ****p < 0.001.

To test the capacity of host DNA depletion with AS, we used five DNA samples primarily extracted with the AllPrep kit and sequenced on Illumina platform using 150 bp PE mode (Figures 2A, 3). We re-sequenced these samples using ONT, with one group serving as a “control group” without adaptive sampling and the other one as a “AS group” with potential reduction or depletion of host DNA reads (Figure 4).

Overall, ONT adaptive sampling yielded 50% less total bases of raw data (average of ∼20′915′948′394 bp in the control group and ∼11′745′064′707 bp in AS group) and 3 times more reads (average of 4′700′719 reads in the control group and 15′331′338 reads in the depletion group) (Figure 4A; Supplementary Table S3). Despite efforts to reduce it, the prevalence of host DNA reads remained relatively high in all samples. However, the length distribution of host DNA reads was restricted to approximately 500 base pairs, suggesting a discernible impact on both the mean read length and the read length N50.

Our assessment of AS efficiency by comparing the host and microbial read numbers revealed a statistically significant, albeit relatively modest depletion rate (between 0.5% and 0.7%) compared to traditional wet-lab-based approaches (Figure 4A). Due to the removal of human reads from the sequencing pool, which comprised the majority of reads in human biopsy samples, sequencing with the AS approach yielded shorter human DNA read lengths. Additionally, the ejection of DNA strands from the pore requires a recovery time, leading to a lower number of reads generated with the AS approach. Consequently, the total output of the AS approach was reduced due to both the lower number and shorter length of reads, while allowing for a substantially greater number of non-human bases to be sequenced in our compartment of interest (Supplementary Table S3; Figure 4A).

Taxonomic profiling revealed AS led to a 2–5-fold increase in the number of reads corresponding to *Escherichia coli* in analyzed samples compared to traditional ONT sequencing without AS (Figure 4B; Supplementary Table S4). Additionally, we observed a modest enrichment of *Bacteroides fragilis* and *Bacteroides thetaiotaomicron* in sample 4AS. In a supplementary analysis, we investigated the efficacy of AS approach in generating better metagenomic assemblies from human intestinal biopsy samples. Our findings revealed that the AS enabled us to assemble more bacterial contigs with greater completeness compared to the non-AS approach (Supplementary Table S5; Figure 4C). Specifically, in sample 4AS, we were able to assemble approximately 57% (3.5 Mbp) of the *B. fragilis* genome with 4X coverage, while only 3% (166 Kbp) of its genome could be assembled from sequence generated without AS (Supplementary Table S5; Figure 4C).

Moreover, our results demonstrated that the greater genome completeness achieved with AS allowed us to recover antimicrobial resistance markers in sample 4AS, which were not detectable in the non-AS sample 4. Specifically, we were able to identify a CepA beta-lactamase and a tetracycline-resistant ribosomal protection protein in sample 4AS. In contrast, these markers were not identified in the non-AS sample 4. AS also enabled the identification of two plasmids in sample 4AS, which were not identified in sample 4 (Table 2). Overall, these results demonstrate the potential utility of the AS approach for the targeted sequencing of specific microbial taxa in complex samples containing high amounts of host DNA and highlight the potential advantages of the AS approach for generating high-quality metagenomic assemblies and identifying important biological features, such as antimicrobial resistance markers and plasmids.

**TABLE 2:** ONT adaptive sampling improves the detection of plasmids. Two plasmids were identified in sample 4 with adaptive sampling but not without AS. Cluster IDs reflect unique taxonomic identifiers for plasmids and are stable over time. *MRF:* Metapair information and *oriT:* origin of transfer.

Plasmid cluster ID	MPF type	oriT type(s)	Median size across samples (bp)	Coverage	Predicted host range	Nearest NCBI accession	Replicon type(s)	Relaxase type(s)
AA336	MPF_F_	MOB_F_	103819 bp	5X	Enterobacterales	LT985217	IncFIA	MOB_F_
AG294	—	—	17450 bp	3X	*Escherichia*	CP019906	—	—

## 4 Discussion

Metagenomic shotgun sequencing of bacterial populations and advanced downstream analysis techniques are powerful techniques to assess the impact of environmental insults or host-derived factors. However, obtaining sufficient bacterial DNA from intestinal tissues can be challenging due to the presence of high amounts of host DNA, which vastly outnumbers microbial DNA. Therefore, substantially decreasing the amount of human DNA is crucial for the successful application of metagenomic sequencing. Host DNA depletion kits are a recent development in the field aiming to enrich microbial DNA in host-associated samples such as blood, feces, urine, saliva, or biopsies. These kits and previous work have advanced a number of strategies; however, a systematic comparison of these approaches alone and in combination has not been done. To address this, we tested host DNA depletion from human intestinal biopsies using i) wet-lab approaches using commercial kits and a protocol inspired by Bruggeling et al. (2021) and ii) a software-based enrichment protocol using a nanopore sequencing platform.

Kit-based microbial DNA enrichment approaches are designed to be effective across most hosts, regardless of the type of microbe present in the sample. They rely on a series of predetermined steps that are optimized to extract the DNA of interest. In contrast, a software-based adaptive sampling method in ONT requires prior knowledge of the host. It is based on the electrical properties of DNA molecules as they pass through tiny pores. The adaptive sampling approach involves selecting specific areas of interest within the host genome and sequencing only those areas in order to identify the areas of interest accurately. Therefore, the key difference between these two approaches is that kit-based microbial DNA enrichment approaches are more robust and can be used across most hosts, while adaptive sampling methods used in ONT require prior knowledge of the host for accurate results. However, the overall efficacy of different assays varied, and some methods yielded acceptable results with up to 28% of host DNA depletion. However, no method depleted 90% of the host DNA, which is required for highly sophisticated analyses such as bacterial genome analyses. Further, potential biases, such as the preferential enrichment of specific microbial taxa remain a concern.

In this study, we first assessed the capacity and performance of various commercially available host DNA depletion kits, such as the HostZERO Microbial DNA kit, the Molzym Ultra-Deep Microbiome Prep Kit, the NEBNext Microbiome DNA Enrichment Kit, and the QIAamp DNA Microbiome Kit for extracting DNA from intestinal biopsy samples (Figure 1). These kits employ different techniques for lysing host cells and enzymatically degrading host DNA. The NEBNext Microbiome DNA Enrichment Kit and QIAamp DNA Microbiome Kit depleted host DNA by up to ∼28% (Figure 2). The NEBNext kit uses a distinct approach by selectively eliminating CpG-methylated host DNA from total DNA extracted using the AllPrep DNA/RNA Mini Kit, and our results confirm the potential utility of this approach. In a previous study with complex respiratory samples, NEBNext also showed an effective host DNA depletion (Thoendel et al., 2016). However, in previous analyses, this method showed poorer results, such as no effective host DNA reduction from saliva samples (Marotz et al., 2018), and relatively low host DNA depletion from resected arthroplasty components and sonicated fluids from prosthetic joint infections (Nelson et al., 2019).

The tested wet-lab-based enrichment methods are not without limitations and biases. Host DNA depletion can introduce a bias toward the identification of specific microorganisms. The kits are designed to remove host-associated DNA and proteins but may also remove microorganisms that are either closely associated with host cells or show DNA characteristics similar to mammalian DNA. This can lead to an underrepresentation of specific microorganisms in the final enriched sample. When analyzing shotgun metagenomic datasets for shifts in richness and taxonomy profile, we observed that all kits, except for the Molzym kit, affected certain bacterial phyla and families stronger than others. Specifically, significant changes were observed in the Actinobacteria, Bacteroidetes, Firmicutes, and Proteobacteria phyla. Within the Proteobacteria phylum, the Pseudomonadales and Enterobacterales orders, as well as the Xanthomonadaceae, Burkholderiaceae, Enterobacteriaceae, and Sphingomonadaceae families, were more enriched after the extraction using HostZero, NEBNext, and QIAamp kits compared to the standard AllPrep kit (Figure 3). The reasons for the selective enrichment or decrease for the mentioned taxa are unclear. On the other hand, the microbiota of the HostZero and QIAamp kits showed a greater diversity of species compared to the standard AllPrep kit. Therefore, these kits have a higher potential to detect bacteria of lower abundance in intestinal biopsies, which might be beneficial in some situations.

A potential alternative to host DNA depletion kits is the use of Oxford Nanopore Technologies’ (ONT) adaptive sampling (AS) feature, which has been shown to increase sequencing depth in bacterial sequences without altering microbial composition when a human reference genome is provided. In fact, recent studies have demonstrated that ONT’s adaptive sampling method reliably increases overall diversity and sequencing depth in clinical metagenomic samples such as ∼113-fold increase in clinical samples of respiratory tract infections (Gan et al., 2021), ∼30-fold enrichment of 148 human genes associated with hereditary cancers (Kovaka et al., 2021), a ∼14-fold increase of the least abundant species in a mock community (Martin et al., 2022), and a 40-fold enrichment of a ZymoBIOMICS mock metagenomic community (Payne et al., 2021). In our hands, adaptive sampling yielded a modest enrichment of bacterial sequences, non-etheless, the overall higher number of bacterial reads enabled a more complete assembly of bacterial genomes and a better identification of bacterial plasmids. However, even with adaptive sampling, complete genomic assembly of even the most abundant species has not been possible, indicating the need for further improvement.

It is worth noting, however, that ONT AS approach also changed the overall identified bacterial composition by depleting some microbial sequences. Particularly, sequencing of the genetic material of the Enterobacteriaceae family, such as *Escherichia coli,* was favoured by this method, similar to the results observed with the DNA depletion kits (Figure 4B). On the other hand, one sample by ONT AS had enriched sequences of two *Bacteroides* taxa, which had not been affected by DNA depletion kits (Figures 4B,C). These observations highlight potential limitations of adaptive sampling, and further investigations are warranted to determine the optimal sequencing approach for different types of microbiome studies. One limitation of our study was that we did not evaluate the wet-lab technique using a host DNA depletion kit and subsequently employing adaptive sampling in ONT. This might help to increase the number of bacterial reads in a tested sample; however, this will also increase the overall sequencing cost per sample. Furthermore, we did not examine the potential usage of formalin-fixed paraffin-embedded (FFPE) tissue, which could serve as a valuable resource for microbial characterization of tissue sections studied previously. However, microbial DNA extracted FFPEs might be affected by various factors, such as damage caused during the fixation and embedding process, and a potentially low quantity of microbial DNA in the sample. Generally, the yield of DNA from FFPE is lower than those obtained from fresh or frozen tissue, which suggests that the tested protocols in this study may not necessarily lead to improved microbial enrichment for FFPEs.

Host DNA depletion up to 30%–50% can be considered an acceptable range for taxonomy classification using shotgun metagenomic approaches. However, it is important to note that having over half of the reads assigned to host DNA still poses a challenge in bacterial genome assembly and identification of SNPs and structural variants. In a recent study, we collected stoma content from the small intestine of six cured colorectal cancer patients who fasted overnight and consumed a standardized breakfast over 6–10 h (Yilmaz et al., 2022). Our results showed that microbial reads obtained from Illumina 150 pb paired-end sequencing increased significantly as time progressed after feeding. However, the sub-strains of the blooming bacteria could only be identified once microbial reads reached over 78% of the total DNA within a given sample. These findings highlight the importance of effective host DNA depletion to enhance microbial read recovery and downstream metagenomic analysis. Based on the previous and current findings, we conclude that current commercial kits have the ability to partially deplete host DNA; however, the depletion rates achieved are not sufficient for sub-strain analysis or structural analysis of the bacterial genomes, even of abundant bacterial species.

In conclusion, the present study provides valuable insights into the efficacy and limitations of host DNA depletion methods for microbiome studies in human intestinal samples. It also highlights the necessity for further development of more effective methods to optimize bacterial DNA yields. With current technologies, researchers designing an analysis pipeline involving microbial DNA enrichment steps must scrutinize several factors when selecting the most suitable approach for their research. Specifically, parameters such as the sample type, the bacterial species or pathogen(s) of interest, cost, and the level of enrichment should be considered. It is crucial to note that the starting proportion of bacterial DNA in a sample highly influences the enrichment factor. Therefore, a sample with lower microbial content will experience a higher fold enrichment than a sample with a higher initial microbial DNA content, provided that an equal amount of host DNA is removed. For instance, it is unfeasible to achieve a 500-fold enrichment if microbial DNA initially constituted only ≤1% of the total DNA (Shi et al., 2022). In these situations, it may be worth exploring the use of alternative technologies for bacterial genome analysis, such as single-cell or long-read sequencing, which may be less impacted by host DNA contamination.

## Data Availability

The datasets generated for this study are available through the Sequence Read Archive NCBI. The Nanopore datasets for this study can be found in the BioProject ID: PRJNA943380 and BioSample: SAMN33717862 (http://www.ncbi.nlm.nih.gov/bioproject/943380).

## Supplementary material

SUPPLEMENTARY FIGURE S1 Diversity differences based on host DNA abundance. The alpha and beta diversity differences were quantified using the Shannon index and the Aitchison distance, respectively, in relation to host DNA abundance. **(A)** The correlation between host DNA abundance and Shannon index was shown for all five kits used in the study with the default setting (left panel), as well as for three kits with lab-optimized protocols (right panel). Each sample is represented by a dot, and statistical significance was observed in both panels (p-value < 0.05). **(B)** The differences in host DNA abundance with Aitchison distance, with the left panel displaying results for all five kits used with the manufacturer’s instruction and the right panel showing results for three kits with lab-optimized protocols.
