# Supplementary figures and images for: Benchmarking microbial DNA enrichment protocols from human intestinal biopsies
Source: Front Genet. 2023 Apr 26;14:1184473. doi: 10.3389/fgene.2023.1184473 (PMC10169731; doi:10.3389/fgene.2023.1184473)

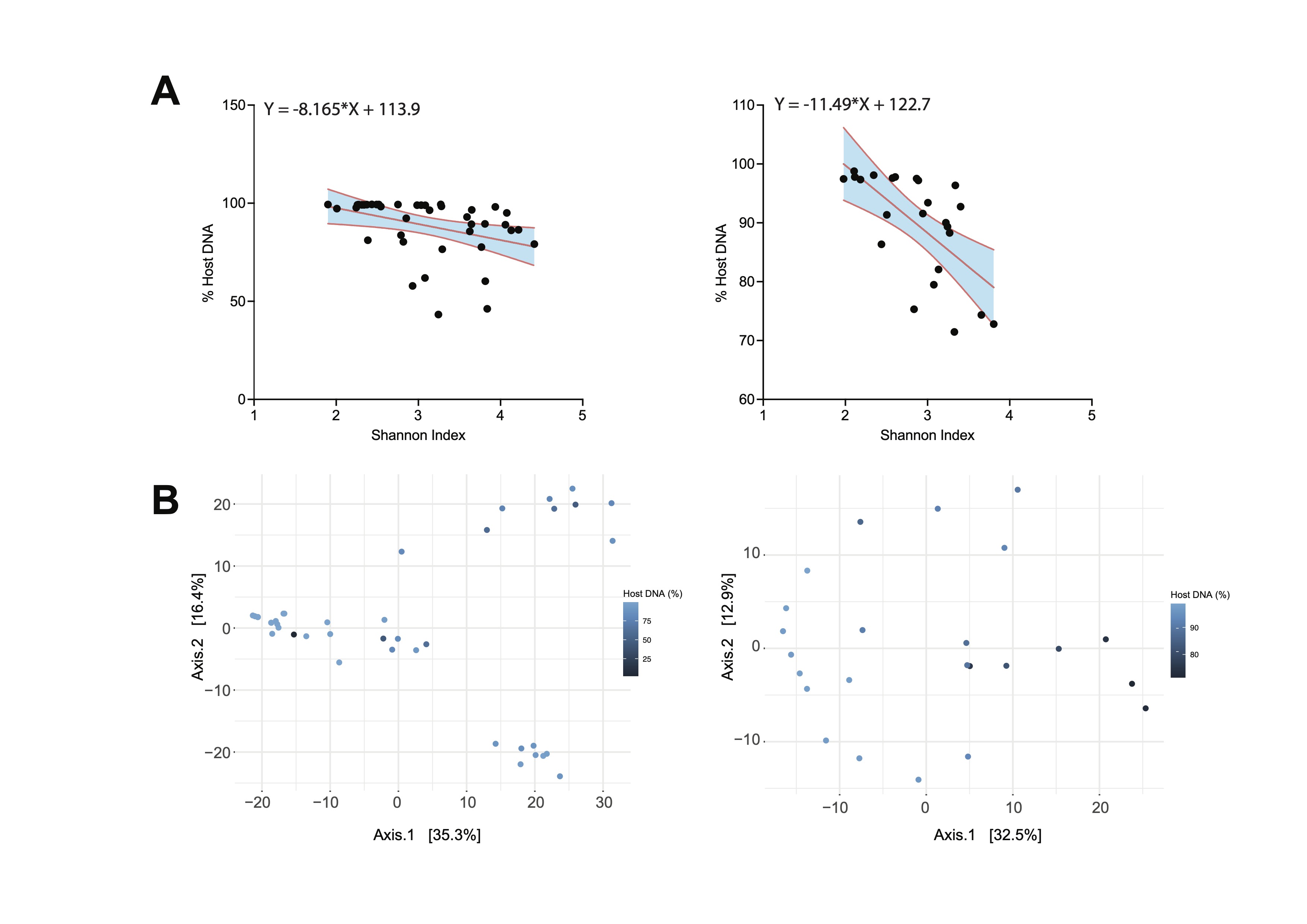

Supplement: Supplementary file 6 [file Image1.jpg]
